# Extracts and Abstracts

**Published:** 1888-08

**Authors:** 


### EXTRACTS AND ABSTRACTS.

### RE-ESTABLISHMENT OF THE MEDICAL PROFESSION.

In the annual address to the Massachusetts Medical Society, June 13, 1888, B. Joy Jeffries, M. D., of Boston, said: It is very generally agreed that the lawyer, the minister, the squire, and the physician, do not hold the same relation to the community as they formerly did. As that position was one of trust and confidence, it well behooves us to carefully study the causes that have broken it down, and correct if possible any fault lying at our door. On the other hand it is equally our duty to right ourselves before the community, if our efforts have again placed us in a position to be trusted and confided in. Moreover, if the community misjudge the whole from a part of the profession, it is the duty of those truthfully striving, not to have their labors misjudged and injured by any false sentiment toward others not so acting.

There are of course many natural causes for the three professions not holding their ancient position. They are now but three of many existing in the world. Science and art and technology have introduced distinctly professional fields of study and occupation.

In the older compact and more isolated communities the diffusion of knowledge was less. The three professions represented nearly the whole of it. Moreover, the diffusion of falsehood was less, though credulity remained. All forms of medical chicanery have increased and widened with the means of reaching by the public press and the post-office a larger circle of the ignorant and credulous, poor and rich. Wealth does not now as formerly include knowledge and common sense. Money is more easily gathered than knowledge, and its accumulation does not preclude the continuance of its owner's previous credulity or gullibility. Moreover, money renders its sudden possessor more dogmatic in his superstition and infallibility. The poor man is open to teaching and advice, the rich man thinks that his wealth places him above both of these.

In the village life of former days any question of medicine, natural science, or natural philosophy was referred to the physician, and his decision taken. The common sense imbibed in medical education and practice enabled him to expose the falsehood started by cupidity and kept alive by credulity. The same confidence was reposed in the minister as to all matters of morality or theology, and in the lawyer as to property and legislation. But we are not concerned with them. Our own position alone should occupy our thoughts and efforts.

Why have the profession as a whole lost their hold on the community and the respect naturally theirs? In this country democracy has brushed away any glamour of office, all that which hedges in a class or an organization. Even that which clings longest, namely any peculiarity of manner or dress which separated us from others, has melted away before the sun of common sense and general intelligence. When the physician possessed more knowledge, more education than those about him in his community, he was respected for these as he should have been. But now-a-days those about him have in the general advance of education gained upon and only too often outstripped him. In the struggle for existence the doctor goes to the level that he is entitled to by his education and the refinement this gives him. If the ranks are filled with men without education, except that which they are supposed to have medically, then the laity finding that such are destitute of the former, very fairly doubt the existence of the latter. Good breeding and a good education are not now as formerly the natural attributes of those legalized by a diploma. Cheap and charity medical schools have multiplied over our country to a greater extent even than seminaries, colleges, and so-called universities. The profession well know that these medical schools are but local advertisements, and that in their struggle for existence they underbid each other, not only as to medical education, but also receive as students and give medical degrees to men comparatively destitute of even a common education. The various State legislatures grant these medical schools the power to issue such degrees, which the laity ignorantly accept as a proof of education, and that is what they are sold and bought for.

Now, in course of time the laity have found out that the holding of a medical degree, being a doctor, does not preclude ill-breeding and lack of education. And they have naturally arrived at the conclusion that a medical man may be pretty low in the social scale, gauged by education, hence that the study and practice of medicine mean but little.

To give every individual in the community, without respect to color or sex, an opportunity for the best education he can attain to and his ability deserves, is a noble charity on the part of the State or the private citizen. For the public or private institution to sanction by its seal ignorance and quackery in any form, is simply cheating the community for the moneyed benefit of the few and unworthy. The poor and unlearned are deluded by the title of doctor, and the delusion has the sanction of the State by the judge on the bench deciding that one medical degree is as good legally as another. The laity know nothing of and cannot judge medical matters, and hence are easily duped into giving State sanction to any and every form of rascality under the guise of medical teaching, from a Druid college to a Christian Scientist's bucket shop, thus helping to fill our grave yards and insane asylums. The community demands protection in everything except precisely where it needs it, namely, from ignorance, prejudice, and superstition.

It is from such causes, it seems to me, that the medical profession as a whole has dropped in the social and educational scale, lost power and lost influence. The public recognize that it is largely entered into as a trade or business, and that many of its members hold degrees and diplomas purchased of the mills, legalized it is true. But so are the dram-shop and saloon legalized. The professors in our law schools well impress on their students that law has nothing to do with justice. One doctor is the same as another to the public to such an extent that even men of attainment and culture fail to distinguish the honorable and well-educated physician from the quack and the charlatan.

I have found many truly scentific men treating us most unfairly in this respect. They tolerate licensed or unlicensed pretenders in medicine without separating them from those of the profession whose knowledge and proved ability really place them on an equality with themselves. They give credence to and put faith in, associate with, men whose ignorance, charlatanism, and want of breeding are such as they never would admit them in their own field of study. Men of science in other branches receive from no class greater recognition and respect than from the best men in our profession, who know enough to appreciate knowledge and its attainment. It is but fair that we ask of them such discrimination as they employ toward each other, and not attribute any protest on our part to narrowness, professional jealousy, etc. It can only be that they as members of the laity imbibe prejudice from inability to discern and judge of medical matters.

The teachers and the examiners in the best medical schools all over the world are demanding greater educational requirements of men desiring to study medicine. And this most rightly, because it is impossible to teach medicine to those without thorough foundation. They cannot learn, and to give them diplomas half earned but adds to the load the educated portion of the profession have to carry. The refinement which education gives is needed in the practice of medicine. Education of a high standard is needed for the doctor to hold his proper relations to the community, besides a thorough knowledge in his calling.

Medicine can only recover its former just position when the world is forced by its existence to recognize that to be a graduate of a good medical school means to have the education of our best universities in addition. The world will not now believe and trust in a man's medical knowledge unless they have proof of other education besides, which adds force and power and refinement. The doctor must be, can be, and will be, a man of science. As such we can ally ourselves with the great body of men striving for knowledge and seeking truth. From the lack of this a large part of our profession is not distinguishable from the man of business or the tradesman, perhaps perfectly respectable, but perfectly commonplace, and worthy only of the position trade and business take in the community.

The avenues to wealth or official power are quite outside our calling. As physicians we cannot compete with trade or business. But this is our common lot with other scientific men. Wealth is a mighty power but it falls palsied before knowledge. Slowly but surely, I believe, is the former yeilding place and respect to the latter. Certainly Crœsus is never more flattered than when treated as possessing knowledge, education, and the refinement it brings.

Are we not in honor bound to do all in our power to bring up the standard of education and the standard of professional requirements in our calling? Educators tell us that it would be a good thing for the world if nine-tenths of our so-called academies and little colleges were swept away and merged in large and strong institutions. Certainly it would be a good thing for the world and for our profession if nine-tenths of the medical colleges were swept away, and what was good in them gathered into a few great medical universities, placed where teaching and study could best be carried out.

Why should not this society quickly raise its standard of requirement for admittance, such as would render membership a proof of educational refinement and professional knowledge? I grant you that moral courage and backbone are needed to do this. But I insist that the possessors of these exist among us, and await only support from the majority. Let them have it, and this at once, or else the best men in the profession must separate and form themselves into a body known and respected for possessing what the others do not. May this necessity never come in my day. But we must sympathize with the man of scientific attainment and educational refinement, forced to recognize another who is ill-bred and half-educated simply because the latter holds also a medical degree which insures nothing of these. That is trades union.

I hear expediency, desire to conciliate for ultimate purposes, compromise, saying, perhaps with bated breath, we cannot be so high toned, we cannot expect doctors to have the refinement of education, or more professional knowledge than enough to get along in practice. I answer at once, with no hesitation or reservation, that there is an increasing number of men in our ranks who have educational refinement and high professional attainments.

Again, I hear it said that the requirements of a physician's vocation tend to break down his literary life and hinder any advance in his medical study. This is but an attempted defence of the ignorant who never should have been allowed to enter our ranks, and who have done so for trade through chicanery. No one of the laity has to descend to the low offices the sacred duty of a physician calls upon him to perform for his patient. Such never detract from his refinement or his dignity. Circumstances may call upon us at times to do any manual or menial work. We elevate it, not degrade ourselves, by performing it as a part of the burden we assume with our title. * * *

Education is the teaching the hands to work at advantage and the brain to think rightly. Certainly this applies to medical education. Proper medical education can not be given the ignorant young man, and only young men should be made doctors. If the hands and brain have not been trained how to work they can not be properly employed in the higher field of human activity occupied by the physician. The advance in modern education comes not from additional amount of facts poured into the school boy or student, but from applying improved systems by which brain work tells better.

It *is* possible to teach the power of observation, deduction, the application of principles, and sharpen the brain, the wits to seize the time for action. The medical schools admitting young men without such preparation, even if they attempt to teach well and thoroughly, never can turn out the best physicians.

The young man in this country who wants to go into medical trade thinks he has only to learn a little and get his diploma as he would fit up a grocery shop. The community thinks so too. The so-called medical colleges scattered over the country are ready to help him for the fees he can scrape together to pay them. This tradesman opens his shop for custom, and the world looks upon and patronizes it like any other shop. But in medicine, as in all the true professions, work but commences with the responsibility of occupation, and never ceases while that lasts. Better for a man if it lasts through his life.

Now these medical colleges, backed by the community, want the profession to foster such trade doctors, and have us accept them as colleagues. These men themselves claim our support and recognition, and would pull us down to their level to help their trade. The time has amply come for this to stop. The physician must rest on his individuality, on his learning and his power of using his knowledge.

If you carefully observe the men teaching and learning in this and the two adjacent buildings,* and then compare them with the men teaching and learning in the building further on, in which we are, or should be, all interested, there will be found a difference of a peculiar and subtle character, the difference between the medical and the technical man. Perhaps only our profession can understand this. I have found very shrewd men in other professions, even the allied ones, much puzzled by it.

Here in technology, arts, sciences aside from us, the student learns facts, physical laws, principles, and their adaptation to physical conditions, and relies on set and fixed laws and rules for action. His work in life is a continuation of this. Here two and two make four, and can always be depended on as making four, mathematically deducible and proved, as we say.

Now in the other building the medical student learns also facts, physical laws, and natural principles. But in practice he has to apply them to unnatural conditions, disease. And he learns that the conditions of disease render mathematical application of seemingly fixed principles impossible. Two and two may not make four, and he must be able to grasp this fact. The study of medicine is for this, and four years is little time enough to learn the needed facts, their application, and, so to speak, misapplication. This it is which besides all else separates us from other professions, and but puzzles them. They see us arriving at results from data that their knowledge and experience prove mathematically can *not* come. This elusive something is the spirit of medicine; he who has it most will most successfully be able to detect and cope with disease, be the most successful physician.

Since the slavery rebellion our nation has settled down to its civilization. Has our profession advanced with increasing education? Not the whole of it, for the reasons I have given. But there are many more men than ever before giving their strength and lives to the accumulation of knowledge in our calling and its diffusion. Never before have there been so many men so highly educated in medicine as now. I cite as proof, the papers and discussions at our society meetings, the articles published in our journals, the respect our best men are gaining from the thorough medical scholars and teachers of England and the continent. I cite further the greatly increasing number of physicians in the various branches of medicine who are becoming good and valuable teachers among us. Never before have we had such competent and thoroughly taught practitioners under thirty years of age. Never before have we had so much true scientific work going on in our profession. The graduates of even our best schools are not competent to stop their work, but seek in Europe the best teachers, to compare their acquirements, and bring back to us the highest medical culture of the old centres of learning.

* Buildings of the Institute of Technology and Natural History Society.

Should these men be classed with the ill-bred and half-educated graduates of the remaining nine-tenths of the medical schools of this country? Because they have the same title, must they be put on the same plane as the business and trade doctors our communites are overrun with? Yet this is precisely the way they are at present treated and regarded by the laity, who make no distinction between one physician and another. And this by all classes of the laity, high and low, rich and poor, learned and unlearned.

The scientific man is often now startled by finding "a doctor" familiar with his own department. It is quite a revelation to him. Why, nearly all the work in the various sciences outside ours, now followed as professions, was formerly done by men, graduates in medicine.

I am free to ask, Is our school and others like it doing *all* needed to fit men to practice medicine, to use their hands and brains professionally? The success and the growing number of polyclinics and post-graduate courses, right among our best schools, seem to me to positively prove that the student and the graduate find there is something more to be learned, and something worth giving time and money for. What better argument for the need of an additional year's study, however this may have to be arranged in reference to the under-graduate department of our universities and colleges?

If there are men who can as teachers attract earnest students outside of the regular courses, I do not see why they cannot be employed as teachers in the schools of four years' curriculum. This extra outside teaching I think has hindered the adoption of a four years' course as compulsory. It has fostered unfortunately the worst form of trade doctors among us, namely, the "two to six weeks'" fully fledged specialist with any "scope."

I disregard the objection to four years on the score of cost. The men who built our present medical school did not stop for this sort of objection, and time has proved them right. I record here my conviction, and I wish I could record the conviction of this society, in the support of the teachers and workers trying to elevate the standard of the profession, and thus, for only thus can it be done, replacing our calling in the respect of the laity, at the same time completely separating us in their judgment from the bands of quacks, trade doctors, *et id genus omne.*

It is perhaps naturally expected of me here to say something in favor of those much *used* and much abused physicians called specialists. Whatever may be said against them, it must be admitted in candor that to stop the work they are doing would check the scientific advancement of the profession as a whole. Specialism means work, seeking science, knowledge, truth. It cannot be said that they keep from the profession or the world the results or benefits of their labors.

The same is now unfortunately true of the specialties as of the general profession. All I have said as to the laity's inability to distinguish between the true and the false applies with ten-fold force as to specialists. Physicians recognize this even in their own inability to decide between pretence and talent, knowledge and ignorance, experienced training and assumed.

I greatly respect other specialties, and cannot see how we can get along without them. I have to thank the men of talent and standing practicing them, and have always found them as willing to help me as I them. * * *

It was said in praise of a physician who died some years ago, that he was never seen in any place his profession did not call him. It was a proof of devotedness to his calling. In the then relations of the doctor to the community perhaps it was wise and necessary. All that has certainly passed away, never to return. We are now forced to be *en rapport* with the world, its people and affairs, and, I believe, with a gain thereby to our usefulness in the practice of our profession. The more a man knows outside of and in addition to his medical work, the better physician will he be. Such knowledge will never hurt his professional judgment, and will very often give him thereby a better opportunity to enforce what he knows to be necessary.

When a patient finds that we are by no means ignorant of *his* special work or business, can express an intelligent opinion thereon and have some interest in it, then that patient is now-a-days much more likely to respect our professional advice and follow it. We have to guide and govern men and women, and we cannot without respect from the governed.

The community somehow still have an idea that physicians as a class are not capable of anything else than their own work, without business capacity, not practical. This certainly is not now the case.

Not only is a physician now-a-days allowed, if I may so say, to have some knowledge of the world of affairs, but he is beginning to be granted some familiarity with literature or with scientific pursuits, even when the latter are not directly connected with medicine. The profession at large and a part of the laity now begin to recognize the value of scientific thought and scientific study in our calling. It does not now hurt a man to be known as scientific—that is, seeking knowledge by mental labor. There was a time when this was a positive detriment, and militated against a man's opportunity to gain a livelihood.

In the great world of scientific work our profession is needed and has its place. Its labor is special and separate, but calls strongly for thinkers, observers, truth-seekers. This is the study of medicine, whilst the practice calls for the greatest endurance, patience, forbearance, toleration, and courage, physical perhaps as well as moral. A thoroughly educated physician is a man of no mean parts, and will be able to hold his own with others in the world's affairs. His training makes him a *gentle*-man.

We should not resent but welcome the coming into the profession of young men with wealth and means that render them independent of work. Even if they practice, they have the right. It was once said with some truth that the possession of thirty thousand dollars would kill any man's advance and work in our calling. That is not true now. But the young men I speak of are most valuable in softening the spirit of gain and strife. There is work in abundance for them, and to advantage of us who must delve for our living. Their position enables them to do for us what we most need but can not accomplish. They have hours for work without anxiety.

To study medicine and take a degree in a first-class medical school after a collegiate course is a training most valuable. The knowledge gained is aside and besides all other, placing the graduated physician at very great advantage over his literary, artistic, and other professional friends. Moreover, the study of medicine is earnest, serious, mentally stimulating, and gives a man breadth of character. It teaches him the value of work. One of the class of young men I am speaking of, whom I had advised to follow our profession, said to me with great satisfaction and self-respect: "To graduate here at our school sickens one for loafing and idleness."

The student learns that life and happiness mean work, work for others or self, but work, without which life at last is found not having been worth the living. The graduate learns also that there is no place in medicine for the Bohemian or the dude; that all attempts to act or imitate the one or the other are wholly out of place, as any of the peculiarities which were the marks of a physician in gone-by days. Even the white cravat worn at other times than when socially demanded, is now pretty well recognized as a medical hypocrisy, Chinese mourning for departed patients. The true physician does and should dress as any other quiet gentleman.

Fortunately for us in this present day, and for the communities in which we live, the absence of means does not preclude the possibility of preparatory and medical education, and that of the highest grade. The State and the individual citizen has now given every man a chance who has brains to use and is willing to use them. It is the State's and individual's *charity*. Harvard University is an endowed educational charity of which every graduate is a recipient.

But absence of means must be an incentive to action, to labor, to study. For myself I know that any good which I have done for the community or for myself has been done from the pressure of complete dependency on my own action. I believe every man finds this the case in life, hard as it may seem to him: Necessity is the mother of invention and the father of success.

In the *Kampf um Dasein* let us join hearts and hands and brains for the re-establishment of our beloved profession.

